# Water Quality of the Mun River in Thailand—Spatiotemporal Variations and Potential Causes

**DOI:** 10.3390/ijerph16203906

**Published:** 2019-10-15

**Authors:** Haoyu Tian, Guo-An Yu, Ling Tong, Renzhi Li, He Qing Huang, Arika Bridhikitti, Thayukorn Prabamroong

**Affiliations:** 1 College of Water Resources and Civil Engineering, China Agricultural University, Beijing 100083, China; tianhaoyu1996@163.com (H.T.); tongling2001@cau.edu.cn (L.T.); 2 Key Laboratory of Water Cycle and Related Land Surface Processes, Institute of Geographic Sciences and Natural Resources Research, Chinese Academy of Sciences, Beijing 100101, China; lirenzhi1990@163.com (R.L.); huanghq@igsnrr.ac.cn (H.Q.H.); 3 Environmental Engineering and Disaster Management Program, School of Interdisciplinary Studies, Mahidol University Kanchanaburi Campus, Kanchanaburi 71150, Thailand; arika.bri@mahidol.edu; 4 Faculty of Environment and Resource Studies, Mahasarakham University, Kantarawichai District, Maha Sarakham 44150, Thailand; thayukorn.p@msu.ac.th

**Keywords:** water quality, spatiotemporal variation, WQI, phosphorus, Mun River basin

## Abstract

The water quality of the Mun River, one of the largest tributaries of the Mekong River and an important agricultural area in Thailand, is investigated to determine its status, identify spatiotemporal variations and distinguish the potential causes. Water quality dataset based on monitoring in the last two decades (1997–2017) from 21 monitoring sites distributed across the basin were analyzed using seasonal Kendall test and water quality index (WQI) method. The Kendall test shows significant declines in fecal coliform bacteria (FCB) and ammonia (NH_3_) in the upper reaches and increases in nitrate (NO_3_) and NH_3_ in the lower reaches. Strong temporal and spatial fluctuations were observed in both the concentrations of individual parameters and the WQI values. Seasonal variation of water quality was observed at each monitoring site. WQI values in August (flood season) were generally among the lowest, compared to other seasons. Spatially, sites in the upper reaches generally having lower WQI values than those in the lower reaches. Excessive phosphorus is the primary cause of water quality degradation in the upper reaches, while nitrogen is the primary parameter for water quality degradation in the lower reaches. Urban built-up land is an important “source” of water pollutants in the lower basin, while agricultural land plays a dual role, affecting across the basin.

## 1. Introduction

Degradation of surface water quality is a critical environmental problem across the world, in particular, in regions with intensive human activities (e.g., intensive agriculture, industrial production, urbanization) which are likely to worsen without the use of effective controls or countermeasures. Much long-term research on different water quality problems, such as land use changes [1], industrial waste water discharge [2], and toxic metal pollution [3,4] have been conducted from a regional to a global scale [5]. Geographical differences and human activities will affect the temporal and spatial variations and trends of water quality [6]. Land use changes and excessive application of nutrients (phosphorus and nitrogen) in agricultural river basins can influence downstream river water quality [7] through the leaching of excess nutrients into water bodies through subsurface and surface runoff. Land use activity significantly influences nutrient loading and discharge, and both annual loss and mean annual concentrations of nitrates are correlated with land use patterns [7,8,9]. Temporal variations in climate, dry and wet seasons or years, also significantly influence nitrogen (e.g., nitrate) fluxes [9]. Land use type, and the resulting loading of nonpoint source discharge of nutrients (e.g., phosphorus and nitrogen) into surface water, can influence the biodiversity of the aquatic environment. Population density also significantly influences the fixed N and P concentrations in river systems [10]. Urban watersheds are major contributors to increased nutrients, with loading rates increasing with the percentage of impervious land area [11]. This is important for today’s rapidly urbanizing river basins which may lead to increased N and P loading.

Based on the measured historical hydrological water quality sequence, statistical analysis methods are used to detect the temporal and spatial changes of hydrological environment elements and influencing factors, which is of great significance to the water environment protection of the basin. Trend detection technology has been widely used in water conditions and water quality variations. Statistical trend analysis makes water quality data to be more comprehensible such that it can provide the scientific guideline to policy decision makers. Two main groups of mathematical tools have been proposed to analyze these trends [12,13], which are parametric methods based primarily on linear and residual models and nonparametric methods, such as the Mann–Kendall test. Though parametric methods are more powerful than non-parametric ones, they require the data be independent and normally distributed. In the cases of data set with a seasonal component or with variables correlated, these methods show false positives [14]. Nonparametric test method hence has better applicability because it has few requirements on data structure. The seasonal Kendall test is an extended form of the traditional Mann–Kendall test. It has strong robustness and accuracy and has been widely used in trend detection of water conditions and water quality sequences [15,16,17].

Water quality assessment is an important aspect of water resource management [18]. Various methods, techniques or models have been proposed to qualitatively or quantitatively evaluate the water quality of rivers or lakes; for example, multivariate statistical techniques such as cluster analysis, factor analysis, principal component analysis [19], discriminant analysis [20], Markov chain models [21], SWAT (Soil and Water Assessment Tool) model [22] and methods based on multi-metric indices [23]. The WQI (water quality index) method, a mathematical method transforming large quantities of water quality data into a single number that represents the general quality of water, has been widely applied to assess both surface water and ground water quality [4,24,25,26,27,28,29,30,31]. The WQI method has many various forms of expression. The differences between the different forms mainly come from two aspects, the weighting methods and the aggregation methods. Different weighting and aggregation methods have a great influence on the water quality evaluation results, and even reached the opposite conclusion [32]. Different WQI methods are suitable for different situation, and also have their own advantages and disadvantages. For example, the NSF-WQI (National Sanitation Foundation-Water Quality Index) method does not represent specific use of water—it represents general water quality. For the Weight Arithmetic WQI method, the number given by water quality index may not be a real indication of the quality of water and a single bad parameter value changes the whole story of the water quality index [33]. However, the WQI reflects water quality conditions with a numerical score, which makes it easy for the public and the policy makers to understand the condition of an aquatic environment [34,35]. In the past ten years, a number of water quality assessment studies have been carried out on various water bodies around the world using the WQI method [36,37,38,39,40].

As the largest tributary of the Mekong River, the Mun River inputs about 20 billion cubic meters of water per year to the Mekong River. Its water quality has an important impact on the water environment in the middle and lower reaches of the Mekong River. The Mun River basin is an important agricultural area in Thailand where human influences such as agriculture and increased urbanization of the several big cities/towns may influence river water quality. The influence of human interventions on the water quality in the basin, however, is currently not well understood. The main objectives of this study, hence, are: (1) to examine the temporal and spatial variation of the representative parameters and general status of surface water quality in the Mun River basin, and (2) to preliminarily investigate the factors which influence water quality in the basin. It is also hoped that present work can be used for reference for other small and medium-sized basins, in particular those have similar natural conditions, being mainly planted in agriculture while undergone rapid urbanization in the Indo-China peninsula. At the same time, the study of the region may shed some light on the management of water resources in the Mekong River Basin.

## 2. Research Area and Methods

### 2.1. Research Area

**Figure 1:**
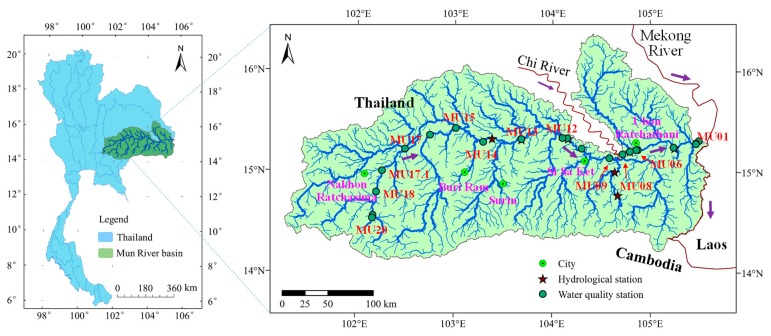
The location of the Mun River basin and the water quality monitoring sites along the river.

The Mun River is situated in northeastern Thailand and is one of the largest tributaries of the Mekong River in Thailand. It lies between latitudes 14° and 16°, and longitudes 101°30’ and 105°30’ and has a length and basin area of approximately 800 km and 71,060 km^2^, respectively [41] (Figure 1). Within the Mun River basin, the terrain is high in the west and low in the east. The dominant soil type in the upper and lower Mun River Basin is sandy and loamy sand, which exhibits good water infiltration and is prone to soil erosion [42]. In the middle of the basin, loamy or sandy loam soils with poor water permeability are predominant [42]. The basin is an important agricultural area in Thailand, particularly for rice planting; about 55%, 14% and 12% of total land area is rice paddy, forest, and crop fields, respectively, which are mainly located in the upper part of the basin [42,43,44].

**Figure 2:**
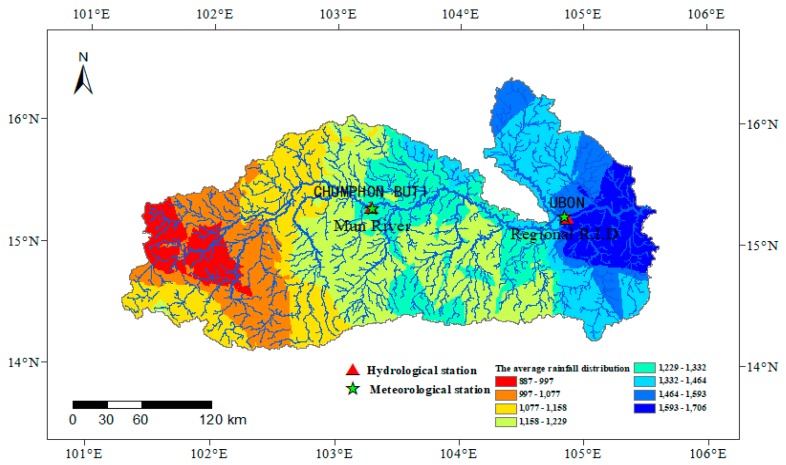
The spatial distribution of annual average rainfall in the basin from 1960 to 2015.

The Mun River Basin has a tropical savanna climate, which is most significantly affected by the tropical monsoons in Asia. The climate and hydrology within the basin show significant seasonal differences brought upon by the seasonal monsoon, causing distinct flood and non-flood seasons, which are from June to October and November to May, respectively. Rainfall in the rainy season accounts for ~90% of the total annual rainfall and rainfall in the dry season only accounts for ~10% of the annual rainfall [42]. The peak rainfall generally occurs in August to September. The spatial distribution is gradually increasing from west (~950 mm) to east (~2100 mm) [7,42,44,45] (Figure 2). The runoff process in the basin also has significant seasonal variations and spatial differences. The runoff in the main flood season accounts for more than 75% of the annual runoff. In the past 35 years (1980–2014), the monthly average flow in flood season and non-flood season in the Mun River Basin fluctuated violently, but there was no significant trend change. According to the analysis of runoff data acquired from the Department of Water Resource of Thailand for year 1980 to 2014, the mean flow discharge of Chumphon Buri station in the flood season (June to October) is 99.6 m^3^/s, which is 2.4 times of the non-flood season (November to May) (Figure 2). The mean flow of the Ubon station in the flood season is 1140.8 m^3^/s, which is 4.0 times that of the non-flood season. The mean annual flow discharge at the Ubon station is 640.7 m^3^/s, almost 10 times of that at Chumphon Buri station in the upper reaches. The peak flow discharge is in general slightly behind the rainfall from September to October. The annual temperature is not lower than 18 °C in the basin [42].

### 2.2. Data Acquisition

Water quality data was acquired from the Pollution Control Department (PCD), Thailand. The PCD has conducted water quality monitoring in the Mun River Basin since 1990s. In order to better reflect the water quality of the river basin, the sites and time of monitoring follow the principle of representativeness, uniformity, and convenience. In total, 21 water quality monitoring sites were distributed along the ~800 km reach of the river (Figure 1). Water sampling and testing were usually conducted four times a year at each site, in February (sometimes in March), May, August, and late November (sometimes in December and even in early January), covering the dry and rainy seasons from 1997 to 2017, and they were conducted at fixed monitoring sites. For monitoring each time at each site, multiple water samples were taken and mean value was obtained after analyzed parameters of the multiple samples. Though the monitoring time was not so strictly fixed, but they were generally comparable since those times basically the same season in each year and covering the dry and rainy seasons. The monitoring in August reflects the water quality during the rainy season, while the monitoring in other three months (February, May, and late November) reflects water quality in the dry season. Typical monitoring sites were chosen for the study on spatiotemporal variation of water quality. The monitoring sites, from upstream to downstream, were: MU17, MU14, MU12, MU09, MU08, MU06, MU03 and MU01, along the main stem of the Mun River. Monitoring site MU17 in the upper Basin receives water flowing through uphill croplands, rice fields, livestock farms and the large city of Nakhon Ratchasima. Monitoring site MU12 is in the middle of the Mun Basin and is predominated by rice fields and rubber plantations, mainly in the Burrirum, Surin and Si Sa Ket. Monitoring site MU06 is in the lower Basin, receiving water from the upper Mun River and the Chi River and discharges from the large city of Ubon Ratchathani. Monitoring site MU01 is at the river mouth connecting the Mun River to the Mekong River.

A large tributary (Chi River) empties into the Mun River just upstream of monitoring site MU08, its runoff and water quality status may have an important influence on the instream water environment of the Mun River. To examine the difference of the instream water environment of the Mun River before and after the confluence of the Chi River, we divide the main stem of the Mun River into upper and lower reaches, of which the upper reaches is the upstream of the confluence of Chi River (i.e., MU09–MU20) and the lower reaches is that downstream of MU09 (i.e., MU08–MU01).

Eight parameters were used as representative indices in the analysis, including dissolved oxygen (DO), biochemical oxygen demand (BOD), total coliform bacteria (TCB), fecal coliform bacteria (FCB), total phosphorus (TP), nitrate-nitrogen (NO_3_-N), ammonia nitrogen (NH_3_-N), and suspended solids (SS). Toxic metal parameters (e.g., Hg, Pb, As, Cd, Cr) were not considered in the current assessment given that the basin is mainly an agricultural area without a mining industry and low levels of industrial production; moreover, the concentrations of toxic metal parameters are generally low, compared to the of guideline values of WHO (World Health Organization) or US EPA (United States Environmental Protection Agency) for heavy metals [4].

Land use data used in this study was acquired from the Department of Land Development of Thailand for years 2000, 2007 and 2015. The land use types were divided into seven categories, which are paddy field—A1, field crop—A2, other agricultural land—A3, forest land—F, miscellaneous land—M, urban and built-up land—U, and water body—W. The association between the land uses and the water quality was later assessed and discussed.

### 2.3. Methods

The seasonal Kendall test [46,47] was conducted to check the trend of concentration of water quality parameters. The seasonal Kendall test is a monotonic trend analysis method based on the Mann–Kendall test and is promoted on this basis, which is a non-parametric test. This method can reduce the influence of data undetected, missed, and singular values on water quality trends by considering only the relative arrangement of data without considering the difference. Compared with regression analysis and other methods, the seasonal Kendall test is not impacted by water quality data during the wet and dry seasons [48]. The seasonal Kendall test is robust and exact, and has been widely used in water quality trend detection [15,16,49].

For the seasonal Kendall test, the null hypothesis $H_{0}$ is a random variable independent of time. Assume that there are n years of water quality observation series $X={\left(X_{1},\;X_{2},\;\ldots ,\;X_{n}\right)}^{T}$, and each subsample *X_i_* contains *P* months data, $X_{i}=\left(x_{i1},\;x_{i2},\;\ldots ,\;x_{iP}\right)$. $Sgn\left(X_{ij}-X_{ik}\right)$ is defined to be zero in case of missing values.

The statistic for each month is: $S_{i}=\sum_{k=1}^{n-1}\sum_{j=k+1}^{n}sgn\left(x_{ij}-x_{ik}\right)$ (1 ≤ *k* < *j* ≤ *n*). For the overall situation of *P* months is $S=\sum_{i=1}^{P}S_{i}$. The number of difference data groups that can be compared during *P* months is *m*:

(1) $$m=\overset{P}{\underset{i=1}{\sum }}\overset{n-1}{\underset{k=1}{\sum }}\overset{n}{\underset{j=k+1}{\sum }}\left|sgn\left(x_{ij}-x_{ik}\right)\right|=\frac{n_{i}\left(n_{i}-1\right)}{2}$$

Under the null hypothesis, we have E(S)=0, $Var\left(S\right)=\sum_{i=1}^{P}Var\left(S_{i}\right)+\sum_{i=1}^{P}\sum_{i=h}^{P}Cov\left(S_{i},S_{h}\right)$, $S_{i}$ and $S_{h}$ are independent random variable functions, so $Cov\left(S_{i},S_{h}\right)$ = 0, the equation is simplified to

(2) $$Var\left(S\right)=\overset{p}{\underset{i=1}{\sum }}\frac{n_{i}\left(n_{i}-1\right)\left(2n_{i}+5\right)}{18}$$

When there are *t* same monitoring values in the *n* years water quality monitoring data, there are:

(3) $$Var\left(S\right)=\overset{p}{\underset{i=1}{\sum }}\frac{n_{i}\left(n_{i}-1\right)\left(2n_{i}+5\right)}{18}-\frac{\sum_{t}t\left(t-1\right)\left(2t+5\right)}{18}$$

The standardized static (*Z*) follows an asymptotical standard normal distribution with mean of zero and variance of one, and is formulated as:

(4) $$Z=\{\begin{array}{ll} \frac{S-1}{\sqrt{Var\left(S\right)}}, & S>0\\ 0, & S=0\\ \frac{S+1}{\sqrt{Var\left(S\right)}}, & S<0 \end{array}$$

The Kendall test statistic is τ=S/m. If *τ* is positive, the water quality observation series has an upward trend. A negative *τ* indicates a downward trend, and the value of *τ* is zero indicates a trend of no change [45]. According to previous studies, the significance level *p* is selected at 0.05 and 0.01 with corresponding *Z* values being 1.96 and 2.58, respectively.

Water quality index (WQI) method is used to comprehensively evaluate the status of the water quality of Mun River. The determination of WQI normally follows three steps: (1) relevant experts, agencies or government departments select representative parameters which have a great impact on water quality; (2) the rating curve and the corresponding fitting equations (see Appendix A Table A1) for each parameter is developed based on experts’ evaluation. The rating curves of the parameters are tested by the PCD to reflect the water quality situation quantitatively and their suitability was adjusted to better determine the real water conditions in Thailand’s rivers [50,51]; (3) according to the impact degree of each parameter on the local water quality, weight for each parameter is determined and aggregation are performed to obtain the final WQI value. The associated equations or scores were then fitted based on these rating curves [51,52,53].

The mean observed values of each water quality parameter were converted into sub-index scores for the monitored water body, respectively, using the fitting equations in Appendix A Table A1. The lowest score and the corresponding parameter for each sample at each monitoring site were determined, and the occurrence (value in percentage) of the lowest scores corresponding to the eight representative parameters during the last two decades (1997–2017) were calculated.

The unweighted harmonic square mean method was used in this study to aggregate sub-index scores. This method allows the most impaired variable to impart the greatest influence on the water quality index (WQI) and acknowledges that different water quality variables will pose differing significance to overall water quality at different times and locations [54]. The formula for the unweighted harmonic square mean is:

(5) $$WQI=\sqrt{\frac{m}{\sum_{i=1}^{m}\frac{1}{SI_{i}{}^{2}}}}$$

where: *SI_i_* represents the sub-index score of water quality parameter i after conversion based on the rating curve corresponding to each parameter, i is each water quality parameter in this study (i.e., DO, BOD, TCB, FCB, TP, NO_3_-N, NH_3_-N, SS), and m is the total number of the selected water quality parameters (m = 8 in this study).

The harmonic square mean *SI_i_* of all analyzed parameters was calculated to obtain the comprehensive WQI value for each monitoring season at each site. Surface water quality is grouped into five classes (Excellent, Fine, Good, Poor, Very poor) in Thailand and their corresponding WQI values are >90, 70~90, 60~70, 30~60, <30, respectively [55].

The establishment of a buffer zone facilitates the spatial analysis of the impact of different types of land use types on water quality. This method is easy to quantify the extent and impact of different land use types on water quality [56]. In order to study the impact of land use change on the water quality of the Mun River Basin, circular buffers of 5 km and 2 km centered with the water quality monitoring sites were established by ArcGIS software (10.2, ESRI, Redlands, CA, USA). The area change of different land use types (i.e., paddy field, field crop, other agricultural land, forest land, miscellaneous land, urban and built-up land, water body) in the basin in 2000, 2007 and 2015 were analyzed. The proportion of the area of land use types in different buffer areas is correlated with the corresponding water quality parameters (DO, BOD, TCB, FCB, TP, NO_3_-N, NH_3_-N, SS). Spearman correlation analysis was performed using SPSS software (22.0, IBM, Armonk, NY, USA) to explore the impact of land use change on water quality over the past 15 years. In addition, seasonal variations of soil nutrients and organic content were assessed to justify its agreement with the water quality. A total of 144 soil samples were taken across the Mun River Basin in February 2017 (dry season) and August 2017 (wet season) and analyzed in the Mahasarakham University’s Environmental Laboratory for available phosphorus (AP) using the Bray II method and total nitrogen (TN) using the Kjeldahl method.

## 3. Results and Discussion

### 3.1. Spatiotemporal Variation of Representative Water Quality Parameters

**Figure 3:**
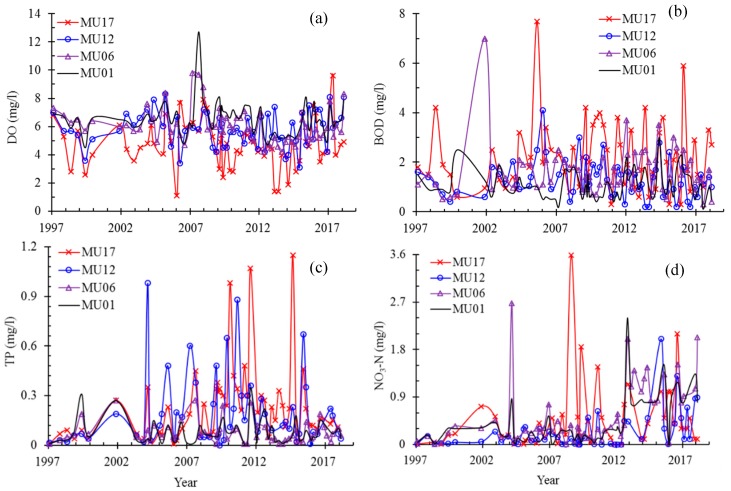
Temporal variation of typical parameters DO (**a**), BOD (**b**), TP (**c**), and NO_3_-N (**d**) at four monitoring sites along the Mun River during the past two decades (1997–2017).

**Figure 4:**
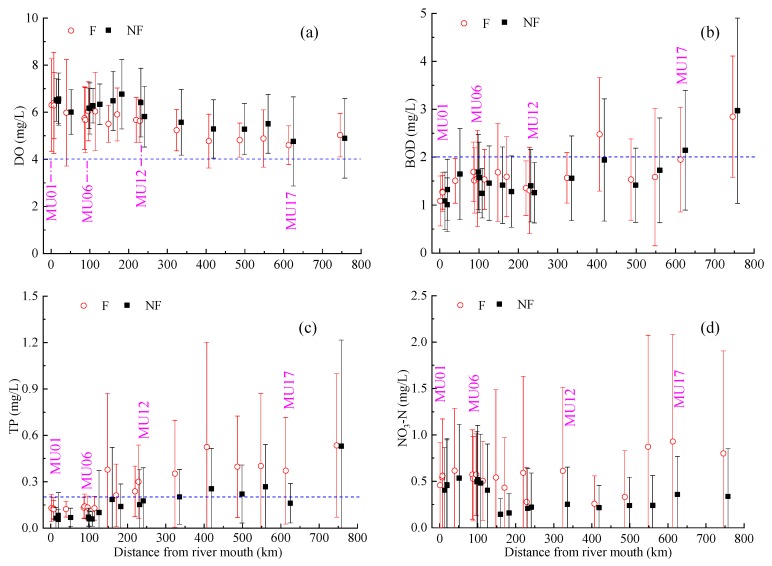
Spatial variation of mean concentrations of typical parameters DO (**a**), BOD (**b**), TP (**c**), and NO_3_-N (**d**) during flood (F) and non-flood (NF) season along the Mun River. The dash blue lines mark the threshold concentration of parameters corresponding to class III of surface water quality standard in Thailand, no line for NO_3_-N since its concentration at all sites lower than threshold value.

**Figure 5:**
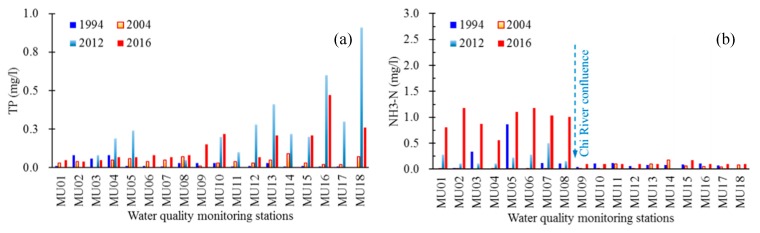
Variation of total phosphorus (TP) (**a**) and NH_3_-N (**b**) values along the Mun River in the dry season (May) of selected years.

The individual parameters showed a large temporally and spatially variability. Marked temporal variations between seasons and over the years were observed at the monitoring sites along the main stem Mun River, during the past two decades (Figure 3 and Figure 4). Spatial differences between sites are also large (Figure 3, Figure 4 and Figure 5). The monitoring sites in the upper reaches (MU09-18) typically have lower DO (Figure 3a and Figure 4a) and NH_3_-N (Figure 5b) and higher concentrations of BOD (Figure 3b and Figure 4b) and phosphorus (TP) (Figure 3c and Figure 4c), compared to the lower reaches (MU01–08). Contrast exists for the spatial variation of NO_3_-N concentrations during flood (F) and non-flood (NF) season (Figure 4d). In flood season the upper reaches typically have higher concentrations of NO_3_-N, while in non-flood season it often has lower concentrations, compared to that in the lower reaches.

The results of seasonal Kendall test of the eight parameters at eight monitoring sites of the upper and lower reaches during the past two decades are shown in Table 1. In the monitoring sites of the upper reaches (MU17, MU14, MU12 and MU09), decreasing trend is apparent for the parameters FCB and NH_3_-N. Contrastingly, in the lower reaches (Monitoring sites MU08, MU06, MU03, MU01), the concentrations of NO_3_-N and NH_3_-N show an increasing trend. No clear trend was found for TP concentrations in different sites (Table 1), although the concentrations in the last decade are higher than those between 1994 and 2004, especially in the upper reaches (MU09–18, Figure 5a).

Statistical analysis revealed sharp contrasts in the concentration of nutrients (TP, NO_3_-N, NO_2_-N, and NH_3_-N) both temporally, between non-flood and flood seasons and spatially, between monitoring sites of the upper (upstream MU09) and lower reaches (downstream MU08) (Table 2). The TP concentrations in the Mun River in the flood season are clearly higher than that in the non-flood season (Table 2, Figure 4c), especially in the upper reaches where the concentration of TP was almost double in the flood compared to the non-flood season; this suggests there is much more phosphorus entering water body during the flood season than in the non-flood season, considering that the flow discharge in flood season is much higher than that in non-flood season. The concentration of NO_3_-N in the flood season in upper reaches is also almost double that in the non-flood season, but its contents in the flood season in the lower reaches does not show a large difference with that in the non-flood season (Table 2, Figure 4d). The contents of NO_2_-N and NH_3_-N also have higher values in the lower reaches than in the upper reaches (Table 2).

The occurrence of the eight parameters with the lowest scores, determined by the fitting equations (details shown in Appendix A Table A1), for each monitoring month (February, May, August, November) at monitoring sites in the upper (MU09–18) and lower (MU01–08) reaches during the last two decades (1997–2017) is shown in Table 3. The higher value (occurrence) corresponding to a certain parameter, the more frequently and further its value is away from favorable values, and the more it negatively impacts the general water quality. The parameters which impacts water quality vary, both spatially (between the upper and lower reaches) and temporally (between seasons) (Table 3). The first three parameters (in italic underline, Table 3) which encumber the general water quality in the upper reaches (MU09–18) are TP, DO and BOD. However, for the lower reaches (MU01–08), the first three parameters change to NH_3_-N, BOD and DO. The primary parameters that influence water quality in the upper and lower reaches of the Mun River are different, though both are nutrient-related (phosphorus and nitrogen).

DO and BOD are both in the top three parameters with highest values of occurrence in both the upper and lower reaches (Table 3), highlighting DO and BOD as important factors which encumber the Mun River water quality. It is noteworthy that SS (suspended solids) concentrations are often high during the flood season, the occurrence of SS with the lowest score ranked forth yearly and second in the flood season (August), both in the upper and lower reaches of the river (Table 3). This suggests there is an association between nutrient pollution and suspended sediment loading, possibly from soil erosion and agricultural runoff, across the Mun River basin. In addition, fecal coliform bacteria also ranked fifth with a high score (9.81) for the lower reaches, but a lower score in the upper reaches (2.34). This could confirm the high influences of human excreta discharges, consistent with high NH_3_-N and BOD and low DO scores, in the lower reaches.

### 3.2. Spatiotemporal Variation of WQI

**Figure 6:**
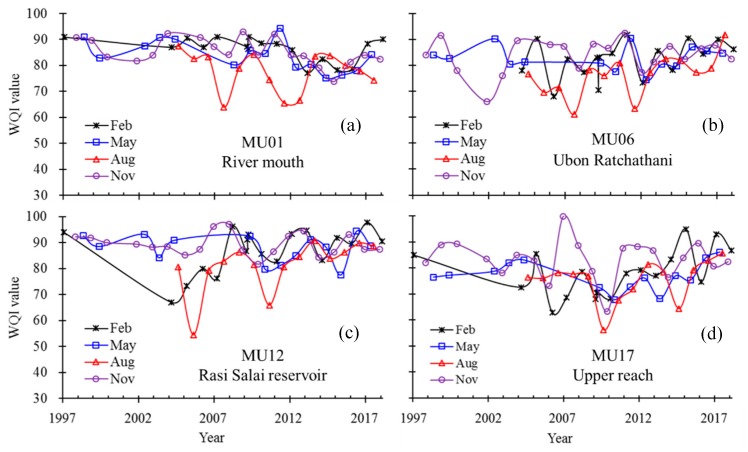
Temporal variations in the water quality index (WQI) values during four monitoring seasons in typical sites MU01 (**a**), MU06 (**b**), MU12 (**c**), and MU17 (**d**) over the past 20 years.

Large spatial and temporal variability of WQI values was observed in the Mun River basin (Figure 6). WQI values of representative water monitoring sites (MU01, 06, 12 and 17) in the basin varied seasonally and yearly. Typically, the WQI values in the flood seasons (August, Figure 6) have the lowest values in a year compared to the values in the non-flood seasons (February, May, and November). However, no clear trends exist for the WQI over the past two decades.

**Figure 7:**
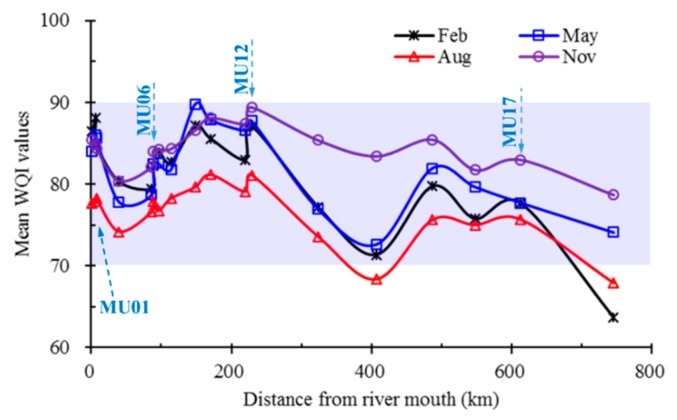
Spatial variation of mean WQI values during different seasons over the past two decades along the Mun River.

Mean WQI values in the same season (February, May, August, and November) during the last two decades shows that the lowest WQI values usually occur during the flood season (August), compared to other monitoring seasons (Figure 7). The spatial distribution of WQI values along the Mun River, based on the 21 monitoring sites in the last two decades, also show considerable variability and it is noteworthy that the upper reaches of the Mun River typically have lower WQI values compared to the lower reaches.

### 3.3. Potential Causes of the Spatiotemporal Variations of Water Quality

#### 3.3.1. Correlation between Land Use Types and Water Quality

**Figure 8:**
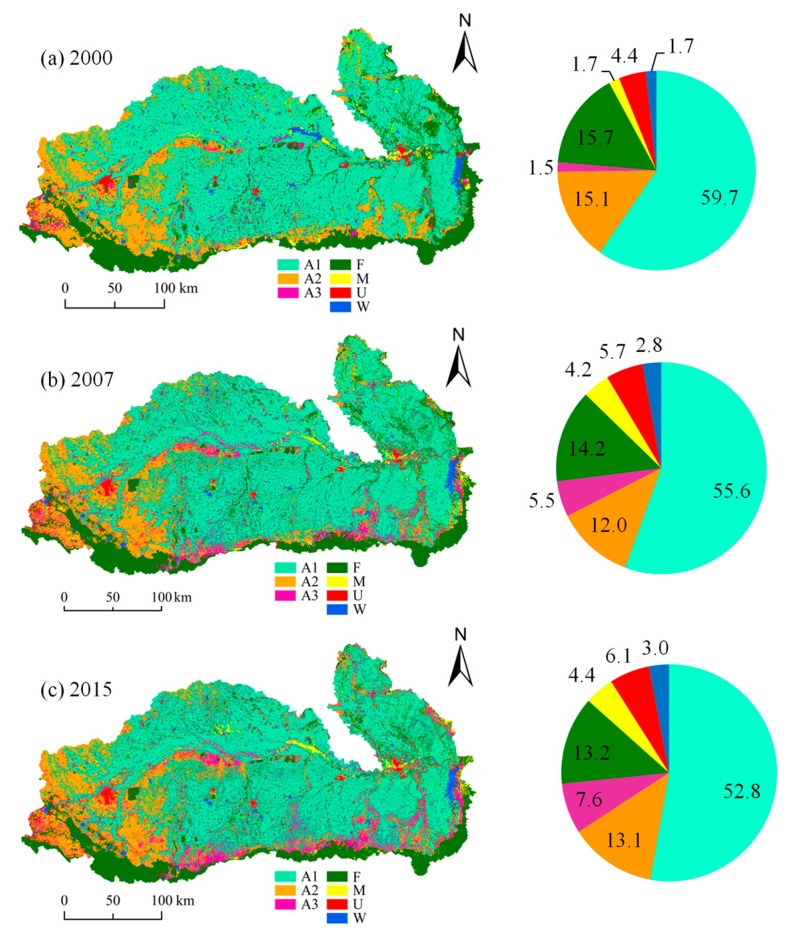
Seven different land use types (left figure) and the corresponding area proportion (in percentage, right figure) in the Mun River basin in (**a**) 2000, (**b**) 2007, and (**c**) 2015, respectively. A1—paddy field, A2—field crop, A3—other agricultural land (perennial crop, orchard, horticulture, etc.), F—forest land, M—miscellaneous land (swamp, saltworks, garbage dump, etc.), U—urban and built-up land, W—water body.

The land use types in the study area were illustrated in Figure 8. The most important land use type in the Mun River Basin is agricultural land, which accounts for more than 73% of the total area of the basin and paddy field is accounting for 96% of this agricultural land area. The area of forest land ranks the second, the urban and built-up land ranks the third, and water body is the smallest. Agricultural land is distributed in the whole basin, of which paddy field mainly distributed in the central and eastern parts of the basin, and field crop is mainly distributed in the western part. The forest land is concentrated in the southern part in highlands. The sum areas of agricultural land and forest land accounts for 92.0%, 87.3% and 86.7% of the total area of the river basin in 2000, 2007 and 2015 (Figure 8), respectively, showing a decreasing trend. In 2015, the area of paddy field and field crop decreased compared with that in 2000, whereas the area of other farmland (including perennial crop, orchard, horticulture, etc.) was slightly increasing. The area of urban and built-up land, miscellaneous land and water body increased

The correlation between the water quality parameters and proportions of the area for different land use types in 2015 on the spatial scales of 5 km and 2 km is showed in Table 4 to explore the impact of land use pattern on water quality. The concentration of TP is positively correlated with the proportion of agricultural land area in the basin (especially paddy field, A1). The correlation is very significant, with coefficient up to 0.69. However, the correlation between nitrogen (NO_3_-N and NH_3_-N) and proportions of agricultural land were very significantly negative, with correlation coefficients up to −0.70 and −0.79, respectively. One of the important reasons is that 70% of agricultural land in the basin is paddy field, having intensive uses of nutrient, both nitrogen and phosphorus, and potentially causing eutrophication in the water bodies [57]. The negative trend between rice field proportion and nitrogen could imply lower contributions of the nitrogen into the water bodies as compared to other competing land use type, such as the urban and built-up lands. Furthermore some scientists report that the rice has strong capacity to absorb nitrogen while weak capacity to absorb phosphorus [58] due to its genetic characteristics. Studies have shown that the nutrient absorption rate of super hybrid rice is N 177.69–189.09 kg/hm^2^, P 36.94–39.80 kg/hm^2^ [59]. However, effects of the nutrient adsorption characteristics for Thai rice fields on low nitrogen discharge into surface water are not reported elsewhere. Further study on nutrient balance in rice ecosystem should be conducted to clarify this finding.

The concentrations of TP and BOD and the proportions of forest land area were negatively correlated, with the correlation coefficients of −0.72 and −0.58, respectively. The correlation coefficient between TP content and forest land in flood season (−0.72) was greater than that in non-flood season (−0.43). Since the rainfall concentrates in the flood season, the forest land is a water-permeable underlying surface, the phosphorus can be retained while dissolving more phosphorus [60]. In addition, the DO content is in a very significant positive correlation with the proportion of forest land (correlation coefficient 0.69), while the SS content is in a negative correlation with it (correlation coefficient −0.48). As such, the decrease in the proportion of forest land had the adverse impacts associated with low DO and high sediment content.

The contents of BOD, TCB, FCB, NO_3_-N and NH_3_-N was positively correlated with the proportion of the urban and build-up land. The correlation was significant for TCB, FCB and NO_3_-N, respectively. In 2015, the correlation between TCB and proportion of urban built-up land had the highest coefficient compared with other land use types, indicating that cities as human activity-intensive areas have an important contribution to the introduction of bacterial pollutants in water bodies. Urban and built-up land represents high-intensity human activities, and domestic sewage contains a large amount of bacterial pollutants such as TCB, FCB and nitrogen nutrients. With the expansion of cities and construction land, the discharge of domestic sewage, municipal sewage, and industrial wastewater has increased correspondingly, which has an important impact on water quality.

#### 3.3.2. Impacts of Confluence from Chi River

The Chi River, the largest tributary of Mun River, has non-negligible impacts on water quality of lower Mun River. The confluence of Chi River, sometimes is the important reason causing obvious difference and even contrasts of concentration of organic and nutrient parameters (e.g., BOD, NH_3_-N, NO_3_-N) between upper and lower reaches of the Mun River. For example, the NH_3_-N values in the lower reaches were unexpectedly high in 2016 (Figure 5b), when a long-term El Nińo episode, which led to drought between summer 2014 to spring 2016 [61], was followed by a major flood in September 2016 due to the tropical storm Rai [42]. According to the water quality report monitored by the Thai Marine Department on 24 May 2016, high organic loading, ranging from 3.30 to 6.71 mg/L BOD_5_ was observed along the Chi River [62]. The poorest water quality was found in the City of Khon Kaen (BOD_5_ = 6.71 mg/L), whose population density is the highest along the Chi River where many industries are located, followed by the City of Kaeng Sanam Nang (BOD_5_ = 5.14 mg/L), where major sugarcane plantations and sugar industries are located [62]. Based on these findings, we assume an association between organics and nutrient loadings and conclude that the high NH_3_-N in the lower reaches can be largely attributed to the discharging of urban domestic and industrial wastewater into the small streamflow during the drought event (non-flood season) from the confluence of the Chi River and cities/towns nearby. The contents of NO_2_-N and NO_3_-N also have higher values in the lower reaches than in the upper reaches, especially in non-flood season (c.f., Figure 4d, Table 2). Apart from raw sewage discharges entering the river from towns or cities, potentially the city of Ubon Rachatani, which is the largest in the lower reaches of the Mun River (Figure 1), the much higher content of nutrient in lower reaches than that in upper reaches could also be from the Chi River’s contribution.

#### 3.3.3. Relationship between Soil Nutrient and Water Quality

**Figure 9:**
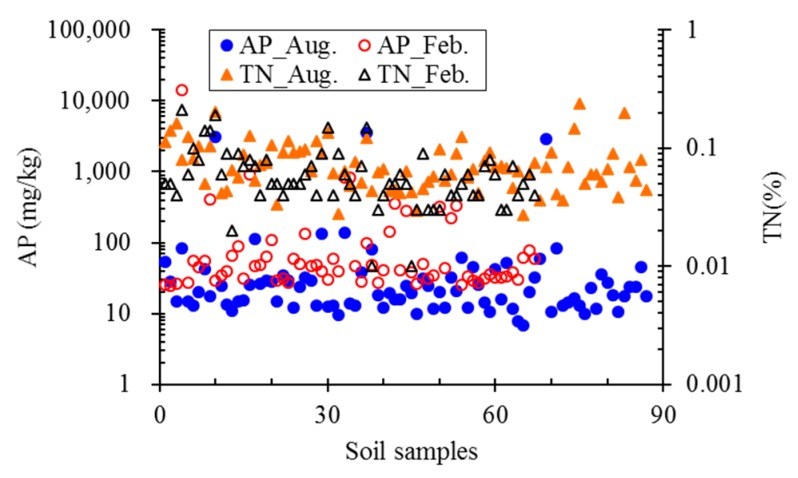
Comparison of available phosphorus (AP) and total nitrogen (TN) contents in soil monitored during rainy (solid symbols) and dry (hollow symbols) seasons in the Mun River Basin.

Basin characteristics, such as drainage density, channel slope, and soil type (lithology, size distribution), influence flow discharge and nutrient transportation from the basin to the water body. Laboratory analysis of available phosphorus (AP) and total nitrogen (TN) contents from soil of the Mun River basin shows that, although there is not a large difference in the TN concentration between the flood and non-flood season, the soil AP content in the non-flood season (February) is higher than that in the flood season (August) (Figure 9). This is in contrast to the TP concentration in the water body. As Figure 4c and Table 2 show, TP concentration in the Mun River was found to be higher in the flood compared to the non-flood season, particularly in the upper reaches, where the difference in TP concentration during the flood season was almost double that of the non-flood season.

Immediately after harvesting in February, fertilizers were applied to crop fields in the Mun River basin, resulting in high AP and TN concentrations in the soil samples. The levels of AP later in the rainy season in August had significantly declined but TN concentrations remained high. This suggests that phosphorus is easily mobilized into the water column and leakage from soil to the water body during the beginning of flood season could quite severely impact water quality.

Our investigation into the water quality of the Mun River is somewhat consistent with other related studies. Cropland discharges large amounts of nitrogen and phosphorus into surface water [7] and agricultural watersheds discharge a higher amount of nutrients than forested watershed [63]. Along with this, row-cropped watersheds export a higher amount of nutrients compared to forests, animal feedlots, and manual storage of fertilizer [9].

For many rivers that are highly influenced by human activities, the water quality during the flood season is better than that in the non-flood season since the higher flows in the flood season lead to a dilution, leading to a reduction in pollutant concentrations. However, in contrast to other rivers (e.g., the Yangtze River in China), the general water quality of the Mun River in the flood season is typically worse than that in the non-flood season. In one hand, this indicates the strong erosion and infiltration effects due to concentrated precipitation and loose soil structures, and the high input rates of nutrients, pollutants, and sediment into the river during the flood season from mainly agricultural and urban runoffs. On the other hand, the decrease in water quality during the flood season could indicate that the Mun River may not be highly influenced by pollutants from point sources, for example, the pollutants discharged from industrial and mining enterprises.

## 4. Conclusions

The contents of the water quality parameters of the Mun River have strong variability, both spatially (between the upper and lower reaches) and temporally (between seasons). The concentration of TP in Mun River in the flood season is higher than that in the non-flood season, especially in the upper reaches. In the lower reaches, an increasing trend in nitrogen concentration (NO_3_-N and NH_3_-N) over the past two decades was observed, particularly during the non-flood seasons. The primary three parameters which impact the general water quality are TP, DO and BOD in the upper reaches, and NH_3_-N, BOD and DO in the lower reaches. Therefore, the primary parameters influencing water quality for the upper and lower reaches of the Mun River are different, although both are nutrient-related (phosphorus and nitrogen).

The general water quality status in the Mun River basin also highly varies, both temporally and spatially. Seasonal variation of water quality existed at each monitoring site along the river. WQI values in August (flood season) are usually among the lowest, compared to other seasons, indicating the worst water quality status. Marked spatial variation in water quality exists from the upper reaches to the river mouth, with monitoring sites in the upper reaches generally having lower WQI values than those in the lower reaches.

Agricultural land area was significantly positively correlated with TP while significantly negatively correlated with NO_3_-N and NH_3_-N. The agricultural land (especially paddy fields) plays a “source” role for the phosphorus load. Urban and built-up land is positively correlated with BOD, TCB and FCB, and most of them are in significant or very significant levels, showing that urban and built-up land plays a “source” role in water pollution load. The water quality of the Mun River Basin is mainly affected by non-point source pollution. Management countermeasures should be taken to control non-point sources of nutrient pollution, with an emphasis on reducing fertilizer application and nutrient inputs into the river.

## Figures and Tables

**Table 1:** The seasonal Kendall test of the eight parameters at eight monitoring sites during the past two decades (1997–2017).

Location	Monitoring Sites	DO	BOD	TCB	FCB	TP	NO_3_-N	NH_3_-N	SS
Upper reaches	MU17	NS	NS	↓ *	↓ **	NS	NS	↓ *	NS
	MU14	NS	NS	NS	↓ **	NS	NS	↓ **	↓ **
	MU12	NS	NS	NS	↓ **	NS	NS	NS	NS
	MU09	NS	NS	↓ *	↓ **	NS	NS	↓ *	↓ **
Lower reaches	MU08	↓ **	NS	↑ **	NS	NS	↑ **	↑ **	↓ **
	MU06	↓ **	NS	NS	↓ **	NS	↑ **	↑ **	↓ *
	MU03	NS	NS	NS	NS	NS	↑ **	NS	NS
	MU01	NS	NS	↑ **	↑ *	NS	↑ **	↑ **	NS

Note: **↓** and **↑** means decrease and increase, respectively; * and ** mean at a 0.05 and 0.01 significance level, respectively. NS means no significant trend.

**Table 2:** Comparison of nutrient contents in the non-flood (NF) and flood (F) seasons in the upper (Upstream MU09) and lower (Downstream MU08) reaches over the last two decades (1997–2017).

Location	Statistical Parameters	TP (mg/L)		NO_3_-N (mg/L)		NO_2_-N (mg/L)		NH_3_-N (mg/L)	
		NF	F	NF	F	NF	F	NF	F
Upstream MU09	Min	0	0	0	0	0	0	0	0
	Median	0.13	0.24	0.11	0.20	0.01	0.02	0.01	0.02
	Mean	0.23	0.46	0.25	0.60	0.06	0.06	0.16	0.11
	Max	3.40	12.80	5.00	10.28	2.11	1.25	8.73	1.28
	cv	1.47	2.44	1.70	1.94	3.09	2.18	4.12	1.80
	cs	5.05	9.74	5.24	5.08	8.78	7.29	9.47	3.09
Downstream MU08	Min	0	0.01	0	0.06	0	0	0.01	0.03
	Median	0.05	0.12	0.26	0.31	0.01	0.01	0.21	0.34
	Mean	0.09	0.13	0.48	0.55	0.15	0.28	0.34	0.43
	Max	6.82	0.35	2.68	2.37	15.40	3.45	2.60	1.51
	cv	4.28	0.53	1.10	0.94	6.24	2.70	1.04	0.73
	cs	16.14	0.70	1.72	1.58	14.44	3.06	1.91	0.98

Note: cv, cs means coefficient of variation and skewness, respectively.

**Table 3:** Occurrence (in percentage) of eight parameters with the lowest value of the eight scores corresponding to each water monitoring month during the last two decades (1997–2017).

Location	Parameters	February	May	August	November	Total
Upper reaches	DO	*4.53*	*5.49*	2.75	*10.99*	***23.76***
	BOD	*6.59*	*6.46*	*3.30*	*6.59*	***22.94***
	TCB	0.69	0.14	0.27	0.69	1.79
	FCB	1.24	0.41	0.14	0.55	2.34
	TP	*9.48*	*5.22*	*9.20*	*6.73*	***30.63***
	NO_3_-N	0.00	0.00	0.82	0.14	0.96
	NH_3_-N	1.37	0.41	0.00	0.82	2.61
	SS	3.98	3.16	*4.67*	3.16	14.97
Lower reaches	DO	2.12	*5.19*	*3.65*	*4.23*	***15.19***
	BOD	*5.77*	*5.00*	2.88	*6.35*	***20.00***
	TCB	2.88	1.15	0.96	1.54	6.54
	FCB	*3.85*	2.50	0.77	2.69	9.81
	TP	0.19	1.92	2.50	1.92	6.54
	NO_3_-N	0.38	0.19	0.00	0.00	0.58
	NH_3_-N	*11.15*	*4.81*	*5.38*	*9.81*	***31.15***
	SS	1.15	0.96	*5.58*	2.50	10.19

Note: TCB, FCB and SS means total coliform bacteria, fecal coliform bacteria and suspended sediment, respectively.

**Table 4:** Correlation between proportions of land use types and water quality parameters in 2015.

Study Scale	Land Types	Period	DO	BOD	TCB	FCB	TP	NO_3_-N	NH_3_-N	SS
5 km	A1	F	0.03	0.30	−0.31	−0.45	0.62 **	−0.59 **	−0.73 **	−0.15
		NF	−0.22	0.14	−0.40	−0.51 *	0.56 *	−0.59 *	−0.56 *	0.36
	A2	F	−0.29	0.07	0.13	0.18	0.28	−0.10	−0.23	0.19
		NF	−0.21	0.21	−0.18	−0.02	0.39	−0.32	−0.19	0.23
	A3	F	−0.05	0.18	0.02	0.26	0.29	−0.33	−0.30	0.28
		NF	−0.08	0.00	−0.26	0.00	0.34	−0.26	−0.35	0.16
	F	F	0.48 *	−0.24	0.09	−0.09	−0.72 **	0.27	0.50 *	0.29
		NF	0.69 **	−0.58 *	0.11	0.24	−0.43	0.21	0.48 *	−0.48 *
	U	F	−0.46	0.29	0.29	0.41	−0.05	0.40	0.14	−0.21
		NF	−0.38	0.51 *	0.59 **	0.48 *	−0.22	0.33	0.04	−0.03
	W	F	0.16	0.07	−0.11	0.08	−0.37	0.22	0.48 *	0.05
		NF	0.02	−0.07	0.04	0.21	−0.51 *	0.46	0.45	−0.43
	M	F	0.25	−0.70 **	0.20	0.20	−0.31	0.44	0.49 *	−0.25
		NF	0.17	−0.16	0.15	0.06	−0.53 *	0.65 **	0.58 *	−0.23
2 km	A1	F	0.27	0.18	−0.14	−0.24	0.69 **	−0.63 **	−0.79 **	−0.14
		NF	0.03	0.05	−0.28	−0.47	0.67 **	−0.70 **	−0.54 *	0.32
	A2	F	−0.19	0.23	−0.22	−0.10	0.00	−0.14	−0.09	0.50 *
		NF	−0.32	0.15	0.05	−0.07	0.44	−0.18	−0.20	0.38
	A3	F	−0.07	0.25	−0.17	−0.02	0.44	−0.61 **	−0.53 *	0.20
		NF	−0.17	0.11	−0.22	−0.15	0.36	−0.31	−0.38	0.39
	F	F	0.38	−0.34	−0.05	−0.14	−0.62 **	0.24	0.35	0.13
		NF	0.49	−0.55	−0.23	0.12	−0.45	0.20	0.36	−0.47
	U	F	−0.16	−0.02	0.49 *	0.27	−0.25	0.63 **	0.37	−0.24
		NF	−0.13	0.40	0.59 **	0.55 *	−0.42	0.55 *	0.24	−0.19
	W	F	−0.03	0.35	−0.26	0.08	−0.26	0.01	0.24	0.09
		NF	−0.05	0.05	−0.01	0.22	−0.24	0.14	0.13	−0.35
	M	F	−0.14	−0.46	0.10	0.28	0.12	0.12	0.08	−0.31
		NF	−0.18	0.12	0.13	−0.08	−0.14	0.34	0.20	0.17

Note: F and NF represent flood and non-flood season; * and ** mean at a 0.05 and 0.01 significance level, respectively.

## Appendix A

**Table A1:** Rating formulas of eight parameters for score converting in Thailand.

Parameter	Concentration Range (mg/L)	Score Rating Formula
DO	0–4.0	15.25 × (DO concentration) + 0.1677
	4.1–6.0	5 × (DO concentration) + 41
	6.1–8.4	12.083 × (DO concentration) − 1.5
	8.5–8.9	755.2 − 78 × (DO concentration)
	9.0–11.2	177.09 − 13.043 × (DO concentration)
	11.3–(≥15.3)	115.68 − 7.561 × (DO concentration)
BOD	0–1.5	100 − 19.333 × (BOD concentration)
	1.6–2.0	101 − 20 × (BOD concentration)
	2.1–4.0	91 − 15 × (BOD concentration)
	4.1–(≥8.8)	56.833 − 6.4583 × (BOD concentration)
TCB	0.0–5000	100 − 0.0058 × (TCB concentration)
	5001–20,000	74.333 − 0.0007 × (TCB concentration)
	20,001–160,000	65.286 − 0.0002 × (TCB concentration)
	>160,000	32.292 − 8 × 10^−6^ × (TCB concentration)
FCB	0.0–1000	100 − 0.029 × (FCB concentration)
	1001–4000	74.333 − 0.0033 × (FCB concentration)
	4001–90,000	62.395 − 0.0003 × (FCB concentration)
	>90,000	32.208 − 10^−5^ × (FCB concentration)
TP	0–1.48	e^(−2.5 × (TP concentration))^ × 100
	>1.48	2
NO_3_-N	0–23	e^(−0.16 × (NO_3_-N concentration))^ × 100
	>23	2
NH_3_-N	0.0–0.22	100 − 131.82 × (NH_3_ concentration)
	0.23–0.50	78.857 − 35.714 × (NH_3_ concentration)
	0.51–1.83	72.278 − 22.556 × (NH_3_ concentration)
	>1.83	42.167 − 6.1024 × (NH_3_ concentration)
SS	0–4	100
	4.1–75	0.0043 × (SS concentration)^2^ − 1.1687 × (SS concentration) + 105.03
	75.1–230	0.0007 × (SS concentration)^2^ − 0.4647 × (SS concentration) + 72.208
	≥230	2
